# Proteomic Approaches in Biomarker Discovery: New Perspectives in Cancer Diagnostics

**DOI:** 10.1155/2014/260348

**Published:** 2014-01-14

**Authors:** Petra Hudler, Nina Kocevar, Radovan Komel

**Affiliations:** Medical Centre for Molecular Biology, Institute of Biochemistry, Faculty of Medicine, University of Ljubljana, Vrazov trg 2, 1000 Ljubljana, Slovenia

## Abstract

Despite remarkable progress in proteomic methods, including improved detection limits and sensitivity, these methods have not yet been established in routine clinical practice. The main limitations, which prevent their integration into clinics, are high cost of equipment, the need for highly trained personnel, and last, but not least, the establishment of reliable and accurate protein biomarkers or panels of protein biomarkers for detection of neoplasms. Furthermore, the complexity and heterogeneity of most solid tumours present obstacles in the discovery of specific protein signatures, which could be used for early detection of cancers, for prediction of disease outcome, and for determining the response to specific therapies. However, cancer proteome, as the end-point of pathological processes that underlie cancer development and progression, could represent an important source for the discovery of new biomarkers and molecular targets for tailored therapies.

## 1. Introduction

Modern molecular methods have in the last few decades paved their way into clinical diagnostic laboratories. The majority of these novel techniques in cancer diagnostic are based on detection of mutations on the DNA level or aberrant gene expression, relying on quantification of mRNA transcripts. Genetic techniques provide information about specific and subtle genetic changes, which have been quite useful in the identification and diagnosis of certain carcinomas, lymphomas, and leukaemia [1]. Compared to the genome or even transcriptome, proteome is much more complex and dynamic. Since proteins are the actual functional molecules in the cell and represent actual conditions, measuring them as a part of the diagnosis could be an advantage in detecting pathological conditions. A myriad of protein biomarkers is already in use in clinical diagnostics (Table 1); however, the methods used for their detection and evaluation are mostly well established techniques, which are decades old, such as serum protein electrophoresis, Western blot, enzyme-linked immunoassays (ELISAs), and a few other immuno-based assays, including methods relying on fluorescence microscopy and flow cytometry. A few of the better equipped laboratories also routinely use liquid chromatography mass spectrometry (LC-MS/MS) for the detection of small molecules, which include amino acids and biogenic amines [2, 3]. MS measurements of proteins and peptides are currently more suited for protein biomarker research, due to technical complexity of the methods, which are not yet suitable for routine diagnostic laboratories, time required to perform the analyses, costs associated with the acquirement of new equipment and training of personnel, lack of thorough analytical and clinical validation of the methods and proteins associated with the particular type of cancer, and finally, problems associated with the sensitivity of the testing for specific rare proteins in protein rich clinical samples, such as plasma, serum, faeces, or saliva [4, 5].

Typical molecular biomarkers currently used in clinical setting are proteins, specific variations in the DNA sequence (germline or somatic), abnormal methylation patterns, aberrant transcripts, miRNAs, or other biological molecules, such as lipids and metabolic products. They can be used to evaluate the progress of the disease and the effects of therapeutic interventions or for estimating cancer risk in individuals with family history of hereditary types of cancers. Protein biomarkers are expected to be reliable predictors of the disease state and clinical outcome, since they probably most accurately reflect the pathogenic phenotype as they are the endpoint of biological processes [6]. However, more high-throughput genetic methods were recently introduced in clinical laboratories than advanced proteomic methods. For example, several gene expression PCR-based or microarray-based tests have been tested for use in clinics and validated in clinical trials, among them MammaPrint, BluePrint, and TargetPrint (Agendia Inc., Amsterdam, The Netherlands) for breast cancer, ColoPrint (Agendia Inc., Amsterdam, The Netherlands), TheraPrint (Agendia Inc., Amsterdam, The Netherlands), and Oncotype DX assay for breast, colon, and prostate cancer (Genomic Health, Inc., Redwood City, CA), Prolaris (Myriad Genetic Laboratories, Salt Lake City, UT) for prostate cancer, ColoGuideEx (Inven2, Oslo, Norway), and ColoGuidePro (Inven2, Oslo, Norway), and ColDX (ALMAC GROUP LTD, Craigavon, UK) for colorectal cancer [7–17]. Studies indicate that these tests could be cost-effective; however, the health insurance systems mostly do not cover their high costs. One of the main problems concerning genetic analyses of gene expression, gene mutations, epigenetic changes, and alterations of miRNA is that only in few cases the functional consequences of gene alterations were resolved. Functional assays, which determine the effect of gene alterations, are immensely complex to design and hard to perform in heterogeneous environment, such as human tissues. Considering that, comprehensive analyses of human proteome from clinical samples, such as serum or plasma, urine, spinal fluid, and tissues, are conducted and publicly available databases as well as bioinformatics analyses relevant to cancer proteomic studies have been recently developed and established [18–21]. Information collected in these large databases enables integration of transcriptomic, metabolomics, and proteomic profiles, which could ultimately contribute to identifying molecular features associated with cancers [19]. The interrogation of data on aberrant posttranslational modifications of proteins could reveal proteins, whose genetic information is intact, but pathogenic alterations render these proteins either nonfunctional, more stable, or more prone to degradation, or they could even obtain the ability to form new interactions with other cellular molecules such as proteins, nucleic acids, lipids, and cofactors, which are not their normal binding partners [4]. These modifications could also be of interest in developing new therapeutic approaches, since they could be reversible depending on the nature of the modification.

Despite remarkable innovations in proteomic methods and technologies in the last years, the integration of new proteomic technologies in clinical laboratories is slow due to the costs associated with acquisition of new instruments, evaluation of biomarker specificity and sensitivity, and obtaining information on clinical validity of biomarkers in large populations. This process is further complicated by the need for training technical and highly educated personnel in novel techniques and interpretation of obtained complex results [22–24]. Implementation of new instruments and methods also requires the establishment of appropriate laboratory reference systems in order to (1) ensure accuracy and reliability of diagnostic measurements; (2) provide quality control standards and standard laboratory procedures; and (3) provide monitoring and quality control assessment of laboratories. Furthermore, even though new high-throughput protein techniques produce large quantities of biological information, very few of the discovered protein biomarkers show the level of specificity and sensitivity necessary for use in clinical setting [22, 25–27].

Deciphering the clinical relevance of candidate proteins or protein profiles and introduction of new protein biomarkers into clinical setting is further complicated by the vast dynamic range of proteins and their normal isoforms, new isoforms associated with the particular type of cancer, aberrant processing into mature forms, anomalous chemical modifications of proteins, formation of immunocomplexes or complexes with other molecules, and heterogeneity of the disease [28]. Certain posttranslational modifications (e.g., phosphorylation, methylation, glycosylation, S-nitrosylation, N-acetylation, lipidation, and proteolysis), which in healthy cells function as a key mechanism to increase proteome diversity, have been found to be altered in tumour cells, rendering nonfunctional proteins or modifying the target locations of the particular protein, and these changes could also be of importance in clinical diagnosis [4, 19, 29]. However, although sensitive proteomic methods have been developed for detecting these changes, translating the diagnostic significance of modified human proteomes in clinical samples is immensely complex [19]. Additionally, modern in-depth analyses revealed evidence of evolutionary dynamics and selective pressures that govern tumour initiation and progression and promote cancer subclonal spatial and temporal heterogeneity [30, 31]. Cancer molecular heterogeneity can be observed on several levels: (1) genetic heterogeneity—copy number variations, point mutations, and different levels of gene expression; (2) heterogeneity in the germline background, which promotes generation of different aberrations in tumour cells and surrounding tumour stroma in individual patients; (3) epigenetic heterogeneity; and (4) phenotypic heterogeneity [30]. These inter- and intratumour heterogeneities add an additional level of complexity. Consequently, the researchers and healthcare personnel meet difficulties in interpreting the results and it is next to impossible to expect that two or more cancer patients could have the same alterations on the protein level.

In this review we first focus on proteomic approaches used in the discovery and validation of new cancer biomarkers. Additionally, we discuss the utility of these methods in research and identification of novel protein biomarkers and in translation of discovered biomarkers into clinical practice. Finally, we review promising biomarkers for most common solid tumours.

## 2. Proteomic Biomarkers in Solid Tumours

The set of proteins encoded by the genome comprises the proteome [32]. It is now well known that the proteome is dynamic and it changes in response to, for example, the physiological status of the organism. The field that encompasses studying such sets is called proteomics. Nonscientific PubMed inquiry finds first review papers using this term dating to 1998. By today, next-generation proteomics has evolved analogous to next-generation sequencing [33]. Modern proteomic technologies enable characterisation of almost complete proteomes and allow a much more in-depth view into biological and pathogenic processes.

Proteomics can be divided into two categories: bottom-up or shotgun and top-down. In the first one, proteins are extracted from a sample, digested with an enzyme (trypsin), and potentially fractionated with liquid chromatography (LC). Peptides are then analysed via tandem mass spectrometry (MS/MS) and proteins in the original sample are identified [34]. In the second one, proteins are also isolated, processed to a limited degree, and, in the end, used for MS analysis intact [35].

A wide variety of separating techniques was developed over the years. Roughly, they can be divided into gel-based and non-gel-based. Two-dimensional gel electrophoresis (2DE) is one of the most widely used gel-based techniques. Dating back to 1970s, it has been the work horse of proteomics for quite some time [36]. As the name suggests, it is used to separate the proteins in two dimensions: according to their isoelectric point (pI) via isoelectric focusing (IEF) and according to their molecular weight via polyacrylamide gel electrophoresis (SDS-PAGE). Proteins, visible as spots on gels, are analysed with an appropriate computer software to identify differentially expressed specimens, which are then excised, enzymatically digested, and identified using MS or MS/MS [37]. Quite a lot of improvements have been made over time, both in the separation (e.g., the invention of immobilized pH gradients) and in the identification department (e.g., the development of MS instruments). A very important upgrade was the development of difference gel electrophoresis (2D-DIGE) [38]. It utilizes the advantage of fluorescent cyanine (Cy) dyes which are used to label the samples prior to any electrophoretic separation. One sample is labelled with Cy3, the other one with Cy5; they are then mixed together and separated in one single gel simultaneously. Scanning under different wavelengths results in two gel images representative of two different sample conditions or states, and at the same time it reduces gel-to-gel variations as the samples are separated on the same gel. Additional advantage adding to the reliability of this method is the use of an internal standard, comprised out of equal amounts of all the biological samples used in the experiment, labelled with a third dye, Cy2.

As already stated, MS can be used to complement a 2DE analysis [39]. Proteins can be identified by means of peptide mass fingerprinting after measuring the mass of peptides obtained by tryptic digestion, as is the case with the traditional matrix-assisted laser desorption/ionization time-of-flight MS (MALDI-TOF MS). Alternatively, peptide mass fingerprinting can be supplemented by sequencing of selected peptides by MS/MS, for example, with MALDI-TOF/TOF or electrospray ionisation MS/MS (ESI MS/MS).

Limitations of the gel-based approaches led to the development of gel-free methods, and MS started to become a core technology of proteomics [33]. In LC MS/MS, proteins are not separated prior digestion; however, peptides are fractionated via LC or other methods at least in one dimension due to sample complexity [39]. A combination of both approaches is also possible, to get a deeper insight into the proteome, such as using a regular SDS-PAGE prior to LC-MS/MS [39].

When using gel-free or combinatorial approaches, one has to choose how the quantitation will be done. Three main options are metabolic labelling, chemical labelling, or label-free methods [40]. In metabolic labelling, such as stable isotope labelling by amino acids in cell culture (SILAC), the isotope-labelled substrate is introduced into every protein during cell growth and division [40, 41]. Chemical labelling is performed by labelling proteins or peptides after their isolation, as is the case with isotope-coded affinity tag (ICAT) and isobaric tags for relative and absolute quantitation (iTRAQ or TMT) [42–45]. Last but not least, label-free methods either directly utilize a peptide's response (intensity) in the mass spectrometer as a quantitative measure or infer quantity indirectly (e.g., spectral counting uses the number of peptide-to-spectrum matches obtained for each protein) [46–52].

Protein microarrays, an approach analogous to DNA arrays, are designed to study protein functionalities in a high-throughput and flexible manner [53]. They are formed by immobilizing thousands of different proteins on a solid surface and can be roughly divided into analytical and functional microarrays. In the first approach, biomolecules with specific binding properties (e.g., antibodies) are printed on the surface to analyse the components of complex biological samples (e.g., serum and cell lysates) or to determine whether a sample contains a specific protein of interest. In the second approach, a large number of individually purified proteins are immobilized and mainly used to comprehensively query their biochemistry properties and activities. A proper surface and immobilization method, as well as signal detection method, which can take advantage of the label (i.e., a fluorescent dye) or be label-free, are important parameters to be considered when using a protein microarray.

Proteomics has facilitated cataloguing of protein profiles in different tissues and biological fluids [4]; however, identification of clinical biomarkers remains one of the most challenging applications [33]. Current biomarkers or biomarker candidates struggle with limited reliability and proper validation as well as with limited sensitivity and specificity [32].

There are several important factors to be considered in the search for a biomarker [54]. First of all, tissues as samples are not easily accessible, and, as they are composed of different cells, they are also very heterogeneous, an issue being addressed with labour intensive laser capture microdissection. However, it can be hardly expected that this approach could be integrated into clinical environment due to its complexity, time consumption, the need for educating the personnel, the costs associated with purchase of equipment, and last but not least, the availability of tissue samples. In clinical diagnosis there has always been a tendency to use most easily accessible specimens for diagnostic procedures, such as blood, saliva, urine, and faeces. On the other hand, regarding the proteomic methodologies, these fluids—although readily available—have the problem of dynamic complexity and are dependent on patient and environmental characteristics. Moreover, collecting the proper number of patients for biomarker validation can also pose a problem. And finally, it is becoming obvious that a single biomarker is not enough for accurate screening or diagnostic purposes; rather a panel of proteins will be necessary [24, 55–57].

### 2.1. Lung Cancer

Lung cancer has been the most common cancer worldwide for several decades and it has also been the most common cause of cancer-related death [58]. Cigarette and other tobacco products smoking is by far the leading cause of lung cancer [59]. Other established environmental risk factors include exposure to second hand tobacco smoke, occupational lung carcinogens (asbestos, nickel, chromium, and arsenic), radiation, and indoor and outdoor air pollution [59]. Older age, acquired lung disease, and infections (i.e., HIV) are also considered risk factors. Fruit consumption, to a lesser extent vegetable consumption, and physical activity are inversely associated with lung cancer risk. Another important factor is positive family history. Certain hereditary conditions, such as Li Fraumeni syndrome and, possibly, Bloom and Werner's syndrome, suggest a possible risk of lung cancer [60]. Mutated *TP53* and *RB1* are also associated with a higher risk, and other candidate genes include cholinergic receptor nicotinic alpha 3 (*CHRNA3*), cholinergic receptor nicotinic beta 4 (*CHRNB4*), and *CHRNA5 *[60], as well as *EGFR* [61, 62].

There are four major histological types of lung cancer: adenocarcinoma, which now occurs most frequently, squamous cell carcinoma, large-cell carcinoma, and small-cell carcinoma [63]. The high number of lung cancer deaths occurs mainly due to the high proportion of tumours diagnosed at an advanced stage [64, 65]. Low-dose computer tomography shows promise for early detection; however, false-positive rates are of concern [63]. A validated, commercially available autoantibody assay *Early *CDT-Lung is also available; it detects autoantibodies against a panel of six tumour-related antigens (p53, NY-ESO-1, cancer-associated antigen (CAGE), GBU4-5, annexin 1, and SOX2) [66, 67]. Furthermore, protein biomarker detection could aid in the diagnostic process. Several potential biomarkers have already been identified (carcinoembryonic antigen (CEA), cytokeratin-19 fragment (CYFRA21-1), neuron specific enolase (NSE), and cancer antigen 125 (CA 125)) and are used in clinical setting; however, few have proven clinical utility, because they are not specific for lung cancer [68].

Intense research in this field gave rise to several review papers covering advancements in the protein biomarker research on several types of samples, such as tissue, blood, pleural effusion, exhaled breath condensate, and urine. In human lung cancer tissue, antioxidant enzyme AOE372, ATP synthase subunit d (ATP5D), 1,4-galactosyltransferase (B4GALT), cytosolic inorganic pyrophosphatase, glucose-regulated Mr 58,000 protein (GRP58), glutathione-S-transferase M4 (GSTM4), prolyl 4-hydroxylase b subunit (P4HB), triosephosphate isomerase (TPI), ubiquitin thiolesterase (UCHL1), isoforms of cytokeratin 7 (CK7), CK8, CK18, and CK19, *α*-enolase (ENO1), pre-B cell-enhancing factor precursor, phosphoglycerate mutase 1, fructose-bisphosphate aldolase A, and guanine nucleotide-binding protein beta subunit-like protein; macrophage migration inhibitory factor (MIF), cyclophilin A (CYP-A), pyruvate kinase M1, manganese superoxide dismutase (MnSOD), peroxiredoxin, proteasome activator PA28, ubiquitin-ligase, prohibitin, Markush macrophage migration inhibition factor (MRP 14), IgE dependent histamine releasing factor, myosin regulatory light chain 2, a-casein; thymosin *β*4 (TMSB4X), acyl-coA binding protein (ACBP), cystatin A (CSTA), cytochrome C, ubiquitin, and desubiquitin showed differential expression [68–71]. Several candidates were identified also in serum, for example, haptoglobin (BH)*β* chain, serum amyloid A (SAA), kallikrein (KLKB1), *α*_1_-antichymotrypsin (ACT), insulin-like growth factor-binding protein 3 (IGFBP3), prostaglandin D synthase (lipocalin-type, L-PGDS), aberrantly glycosylated apolipoprotein C3 (ApoC3), highly fucosylated forms of complement component 9 (C9), retinol binding protein (RBP4), *α*_1_-antitrypsin, squamous cell carcinoma antigen (SCCA), nectin-4, and pentraxin-3 [68, 71, 72]. In plasma, differential expression was observed for lung surfactant protein SFTBP, WAP four-disulfide core domain protein 2 (WFDC2), and angiopoietin-related protein 3 (ANGPTL3) [71]. Another sample source for potential biomarker candidates is pleural effusion, where Niemann-Pick disease type C2 protein (NPC2), periostin, multimerin 2, CD166, and lysosome-associated membrane glycoprotein-2 (LAMP-2) were differentially expressed [68, 73]. VEGF, bFGF, angiogenin (ANG), TNF-*α*, and IL-8 were altered in exhaled breath condensate [71], and HP, calprotectin (composed of S100A8 and 9), and zinc-*α*2-glycoprotein (AZGP1) in saliva [68]. Last but not least, urine analysis also enabled identification of CD59 glycoprotein, transthyretin, G(M2) activator protein (GM2AP), and Ig-free light chain as potential biomarker candidates [69].

Other recent efforts to discover protein biomarkers associated with lung adenocarcinoma include a 2D-DIGE and MALDI-TOF analysis, where Zhou et al. identified 22 differentially expressed proteins in tissue samples. Increased levels of tyrosyl-tRNA synthetase (TyrRS) and microtubule-actin cross-linking factor1 (MACF-1) were confirmed using immunohistochemistry and serum ELISA [74]. Similar basic approach was utilized by Tan and colleagues who identified and verified via Western blot and immunohistochemistry isocitrate dehydrogenase 1 (IDH1) as a potential diagnostic and prognostic biomarker [75]. With iTRAQ, anterior gradient homolog 2 (AGR2) was identified as overexpressed in lung adenocarcinoma tissues [76]. An interesting approach was used by Gámez-Pozo et al.: after phosphopeptide enrichment they analysed paired non-small-cell lung cancer tissue samples by LC-MS/MS. PTRF/cavin-1 underexpression and migration inhibitory factor (MIF) overexpression were confirmed using Western blot and immunohistochemistry [77]. Differentially expressed proteins were determined also by reverse-phase protein array: among others, caveolin 1 and collagen type VI were underexpressed, while cyclin B1, ACC-pS79, CHK2, and IGFBP2 were overexpressed in lung cancer tissues [78]. The finding was validated with Western blot. Zeng and colleagues used laser-capture microdissected tissues and analysed them with iTRAQ [79]. Three proteins, GSTP1, heat shock protein beta-1 (HSP27), and creatine kinase brain-type (CKB), were validated using Western blot and immunohistochemistry, and the authors proposed them as potential biomarkers for early detection of lung squamous cell carcinoma. MALDI-TOF on bronchoscopic biopsy samples is another approach for potential biomarker identification. In this manner, calcyclin was identified as being under-expressed in small-cell lung cancer and verified with immunohistochemistry [80]. Immunohistochemistry was also used to determine differential expression of endothelial cell protein C receptor (EPCR), as well as increased metalloproteinase-2 (MMP-2) and reduced *β*-catenin levels, which may play important roles in initiation, progression, and metastasis of non-small-cell lung cancer [81, 82]. In sera of patients with lung cancer, especially in those with squamous-cell carcinoma, cytokeratin 2G2 was elevated [83]. In another study, protein profiling using ProteinChip and MALDI-TOF revealed that fibrinogen alpha chain was elevated in the sera from patients with stage I squamous-cell carcinoma [84]. Serum activated protein kinase C (PKC*α*) was also proposed as probable biomarker applicable to lung cancer diagnosis [85]. Liu et al. performed LC-MS/MS on serum samples in order to identify proteins associated with non-small-cell lung cancer [86]. Immunohistochemical staining on a tissue microarray was then carried out for alpha-1B-glycoprotein (A1BG), leucine-rich alpha-2-glycoprotein (LRG1), ubiquitin carboxyl-terminal hydrolase 1 (USP1), and mucin-5B as candidate biomarkers. Their levels were significantly elevated in lung cancer tissue. A1BG levels were also determined as significantly elevated with Western blot on sera samples. Another candidate for a cancer specific single marker capable of identifying early-stage lung cancer within at-risk groups without resort to invasive procedures is a variant form of the nuclear matrix-associated DNA replication factor Ciz1 [87]. Its clinical utility was inferred from Western blot on two independent larger sets of plasma samples. Bronchoalveolar lavage fluid is another sample type that could be used for candidate biomarker searching. Pastor et al. used it for 2DE and MS analysis of several groups of samples, including lung cancer group and control group. They identified seven differentially expressed proteins in the tumour versus control setting and confirmed increased AKR1B10 levels using Western blot [88].

### 2.2. Breast Cancer

Breast cancer is by far the most common cancer in women and second most common in both sexes [58]. It ranks as the fifth cause of death from cancer overall. Besides female gender and age, obesity, diet, and physical activity have been linked to breast cancer [89, 90]. It is now known that hereditary breast cancer accounts for 5–10% of all cases. The first discovered susceptibility genes were *BRCA1* and *BRCA2*, but further studies revealed other genes such as *TP53*, *PTEN*, *STK11*, *CHEK2*, *ATM*, *PALB2*, *BRIP1*, and *CASP8* [91]. To date, breast cancer risk assessment is largely restricted to testing for high-penetrance mutations by genetic methods.

Invasive breast cancers constitute a heterogeneous group of lesions. Most of them are adenocarcinomas and the most common types are ductal and lobular [90]. Mammography is the primary imaging modality for population-based breast cancer screening and early detection does decrease breast cancer mortality [92]. However, mammography remains an imperfect test, and it does not detect all breast cancer types.

Based on prognostic factors and hormone receptor status, as well as the extent of surgery performed, adjuvant treatment may be given that includes hormone manipulation and/or chemotherapy and local radiotherapy [90]. However, even with advances in surgical and adjuvant therapies for early-stage disease, most patients eventually experience disease progression or recurrence [91].

There is no molecular biomarker sufficiently powered for use in current clinical practice for breast cancer screening or early detection [93]. The mucin glycoproteins MUC-1 and cancer antigens CA 15-3 and CA 27-29 are the best characterized serum markers related to breast cancer, but they have not been recommended for diagnostic use due to low sensitivity [94]. New potential biomarkers are being sought and recently several review papers summarized some of the candidates found in tissues, blood, nipple aspirate fluid, ductal lavage, pleural effusion, fine-needle aspiration, or core needle biopsy [93, 95, 96]. In serum, isoform 1 of inter-alpha trypsin inhibitor heavy chain (ITIH4), fibronectin 1, CXCL9, apolipoproteins ApoA1, ApoA2, ApoC1, ApoC2, ApoC3, and ApoE, C3a des-arginine anaphylatoxin (C3adesArg), C3f, C4a, platelet factor 4, haemoglobin *α*-chain and *β*-chain, transferrin, epidermal growth factor receptor (EGFR), mammaglobin, afamin, *α*-2-macroglobulin, ceruloplasmin, bradykinin, transthyretin, fibrinopeptide A, and fibrinogen *α* showed differential expression [93, 96]. Gross cystic disease fluid protein 15 (GCDFP-15), *α*1-acid glycoprotein (AAG), and basic fibroblast growth factor (bFGF) were differentially expressed in nipple aspirate fluid [93]. Identified and validated as differentially expressed in tissue samples were also ubiquitin, protein S100-A8, *α*-B-crystalin, HER3, cathepsin H (CATH), heat shock protein beta-1 (Hsp27), protein S100-A6, and desmoglein-3 (DSG3) [95].

S100 proteins are a potentially promising group of biomarkers in cancer development and progression. A large-scale proteomic screening using 2DE and MS for identification and Western blot for validation identified several S100 proteins (11 isoforms as 7 members) as overexpressed in breast cancer tissues [97]. Similar methodology was used to identify over-expression of calreticulin [98]. Using SDS-PAGE, LC-MS/MS, and Western blot protein disulfide isomerase A3 (PDIA3) was identified and validated as overexpressed in breast cancer tissue [99]. A-Kinase Anchor Protein 4 (AKAP4) was also determined as overexpressed in breast cancer tissues using immunohistochemistry and ELISA assay showed that anti-AKAP4 autoantibodies were elevated in sera of patients, making them a potential biomarker for early detection and diagnosis [100]. Metastasis-associated in colon cancer-1 (MACC1) was found overexpressed by Western blot and immunohistochemistry in breast cancer tissues [101]. Moreover, it was associated with survival and as such showed potential as a prognostic biomarker. Similarly, over-expression of lysosome-associated protein transmembrane 4 beta (LAPTM4B) was determined immunohistochemically, and it correlated with disease progression and poor prognosis [102]. Breast cancer tissues were also found displaying over-expression of *γ*-glutamyl hydrolase (GGH) and fatty acid amide hydrolase (FAAH) and underexpression of TAF5-like RNA polymerase II p300/CBP-associated factor (PCAF)-associated factor 65 kDa subunit 5L (TAF5L) [103]. Bone morphogenetic protein 6 (BMP6), on the other hand, was under-expressed in breast cancer tissues [104]. Its differential expression correlated with the oestrogen and progesterone receptor status, tumour grade, and enhanced proliferation. Underexpression was also determined for huntingtin-associated protein 1 (HAP1) [105]. Using sera as samples and multiple fractionation steps (protein depletion, lectin affinity fractionation, IEF separation, and LC-MS analysis), the following candidates were selected as breast cancer-associated proteins: thrombospondin-1 (TSP1) and 5 (TSP5), alpha-1B-glycoprotein (A1BG), serum amyloid P-component (SAP), and tenascin-X (TN-X) [106]. SAP and TSP5 were increased in breast cancer serum, A1BG showed a pI shift and a slight increase in total abundance in the cancer samples, TSP1 showed changes in glycan structure, and TN-X was both increased and showed glycan structure changes. The slight over-expression of the latter was also verified with ELISA. With 2DE, MS, Western blot, and ELISA inhibitor of apoptosis protein-like protein-2 (ILP-2) was determined and validated as overexpressed in sera of breast cancer patients [107]. Biotinidase (BTD) is another potential biomarker. Kang and colleagues identified it using ICAT and validated its underexpression with Western blot in plasma samples from breast cancer patients [108]. Another study using plasma showed that thrombospondin-1 (THBS1) and bromodomain and WD repeat-containing protein 3 (BRWD3) were overexpressed in breast cancer using mTRAQ and Western blot [109]. In a 2D-DIGE and Western blot experiment, Zhang et al. identified several differentially expressed proteins in saliva of breast cancer patients and healthy controls: carbonic anhydrase 6 (CA6) showed the most significant difference among four validated proteins [110]. An interesting approach was used by Bohm et al. They analysed tears of breast cancer patients and compared them to normal controls using SDS-PAGE and MALDI-TOF/TOF [111]. They analysed over 20 differentially expressed proteins, such as C1Q1, ALDH3A, or TPI, but they require further validation.

### 2.3. Prostate Cancer

Prostate cancer is the second most frequently diagnosed cancer worldwide and the sixth leading cause of cancer death in men [56, 112]. A familial history of prostate cancer, increasing age, ethnicity, low testosterone levels, diet rich in fats, and *BRCA1/2* mutations can contribute to the development of this neoplasm [56, 113–115]. GWAS studies and large-scale population screening studies revealed several other susceptibility loci, which include genes *HPC1*, *HPC10*, *HPC14*, and *TERT* [116–119].

Prostate specific antigen (PSA) has been the mainstay for diagnosis and prognosis of prostate cancer in blood [120]. The routine use of PSA screening remains controversial, owing to its limited specificity and sensitivity. Its usefulness is limited because there can be other different reasons for elevated PSA levels, including routine rectal examination of prostate, benign prostate enlargement, inflammation, infection, age, race, and normal leaking of PSA in the circulation [120, 121]. One advantage of detecting PSA levels is that it is tissue specific, so a rise of its concentration in blood is fairly specific to a prostate problem. However, PSA fails to discriminate between aggressive tumours and low-risk ones and between malignant disease and other benign prostate conditions, and as such, overdetection and overtreatment represent critical consequences of PSA-based screening [122–124]. For these reasons, it is imperative to understand the underlying molecular mechanisms leading to prostate cancer in order to identify more sensitive and specific biomarkers to enable more accurate diagnosis and prognosis [56, 120].

A number of proteomic studies using different MS approaches, immunohistochemistry, ELISA, RIA, and bioinformatic analyses attempted to identify screening/diagnostic (protein biomarkers used for the detection of cancer), prognostic (protein biomarkers, which are used to predict the course of the disease), and stratification (proteins that predict the response to treatment modalities) protein biomarkers in tumour tissues (extensively reviewed in [56, 125]).

Ideally, however, for early diagnosis of the malignant disease, the detection of prostate cancer biomarkers should be based on screening procedures in serum, plasma, urine, prostatic secretion, or seminal plasma, since collecting these biological fluids is minimally invasive and fast [56]. Rehman and colleagues profiled pooled serum samples from 4 carefully selected groups of patients representing the various stages of prostate cancer development and progression using a 4-plex iTRAQ approach [126]. They identified 75 proteins, which belonged to diverse biological pathways such as protein metabolism and modification; blood clotting; proteolysis; immunity and defence; complement mediated immunity; and blood circulation and gas exchange. Interestingly, some of these have previously been reported as candidate prostate cancer biomarkers, including CRP, alpha-2-macroglobulin, ceruloplasmin, zinc-alpha-2-glycoprotein, beta-2-microglobulin, and fibronectin [126–131]. Theodorescu et al. speculated that relevant quantities of prostate cancer biomarkers could be present in prostatic fluid, which is preferentially secreted in void urine [132]. Using CE-MS they identified a panel of 12 urinary polypeptide markers and later validated these markers in a blinded prospective multicentre study on a larger set of patients. Prostate cancer was detected with 89% sensitivity and 51% specificity. Including age and percent free PSA to the proteomic signatures resulted in 91% sensitivity and 69% specificity. Polypeptide markers were identified by sequencing and several different proteins were identified, including sodium/potassium-transporting ATPase *γ*, collagen *α*-1 (III), collagen *α*-1 (I), psoriasis susceptibility 1 candidate gene 2 protein (also called SPR1), fragments of glioma tumour suppressor candidate region gene 1, hepatocellular carcinoma associated protein TB6, histone H2B, osteopontin, polymeric Ig receptor, transmembrane secretory component, prostatic acid phosphatase, prostate specific antigen, fibrinogen alpha chain precursor, and semenogelin 1. Interestingly, the majority of marker candidates in this study were determined as downregulated in patients with prostate cancer compared to patients with negative biopsies. Similar study was later performed in an independent cohort of 184 patients in Germany [133]. They confirmed the utility and applicability of the test for routine clinical practice, obtaining a high negative predictive value of 92%. A negative UPA-PC test result in patients with slightly to moderately increased total PSA could initiate a specific monitoring programme for patients, including regular PSA examinations rather than examination of prostate biopsy [133]. Overall the test showed a sensitivity of 86% and specificity of 59%. Quantitative proteomic analysis using collagenase digestion of tissue samples followed by glycopeptide-capture MS/MS revealed 5-fold over-expression of CD90 glycopeptide in cancer versus noncancer tissues. Furthermore, three differently glycosylated forms of CD90 were observed. CD90 (cluster of differentiation 90), also known as Thy-1 (thymocyte differentiation antigen 1), is a N-glycosylated cell surface protein first identified in the thymus as a T-cell maturation and differentiation marker. Immunohistochemical analysis of prostate cancer samples showed distinct and differential overexpression of CD90 in cancer-associated stroma compared with noncancer tissue stroma. Since CD90 might be released from cells, the authors then attempted to identify CD90 in void urine samples and detected CD90 peptides by ICAT in the preprostatectomy samples but not in the postprostectomy samples, confirming that CD90 is secreted by prostate cancer stromal tissue. PEDF (pigment epithelium-derived factor) and zinc-alpha2-glycoprotein were identified using 2D-DIGE followed by LC-MS/MS in a small sample of patients with different grades of prostate cancer [134]. Both proteins were extensively validated in a larger independent cohort of patients, and the results indicated that PEDF is an accurate predictor of early stage prostate cancer. PEDF was also studied in patients with high-grade prostatic intraepithelial neoplasia, which is most likely precursor of prostate cancer, and patients with prostate cancer, revealing that PEDF could be significant predictor of prostate neoplasia. All of the patients with prostate cancer had weak expression of PEDF, 2 patients with high-grade prostatic intraepithelial neoplasia showed strong PEDF expression, 3 patients had moderate expression, and 6 patients with high-grade prostatic intraepithelial neoplasia weakly expressed PEDF. The ten-month follow-up study demonstrated that 2 of 6 patients with high-grade prostatic intraepithelial neoplasia with weakly expressed PEDF subsequently developed prostate cancer. Several other studies identified different protein biomarkers in sera or urine, including afamin, CXCL16, spondin 2, pentraxin 3, engrailed-2, fibronectin 1, eukaryotic translation elongation factor 1, interleukin-6, ceruloplasmin, and complement protein C5; however, further studies are needed to confirm their role in prostate carcinogenesis and their usefulness as clinical biomarkers [126, 130, 135–137].

### 2.4. Colorectal Cancer

Colorectal cancer is the second or third most commonly diagnosed cancer in men and women in the world, respectively. The major disease pathways include the aneuploidy or chromosomal instability pathway involving mutations in *APC*, *DCC*, *TP53*, *KRAS*, *SMAD2,* and *SMAD4* and the CpG island methylator phenotype (CIMP) pathway, which is the second major pathway leading to the development of sporadic colorectal cancers and includes sporadic microsatellite instability (MSI) high cancers. The third pathway, the MSI pathway, is the consequence of germline mutation in a DNA mismatch repair (MMR) genes (i.e., *MLH1*, *MSH2*, *MSH6*, and *PMS2*) [138, 139]. The hereditary deficiency in MMR genes is the cause for the most common form of hereditary colorectal cancer, Lynch syndrome (previously known as hereditary nonpolyposis colorectal cancer), accounting for 1% to 3% of all colorectal tumours [140]. *MUTYH*-associated polyposis (MAP) is an autosomal recessive hereditary syndrome that predisposes individuals to attenuated adenomatous polyposis and colorectal cancer [141–143]. It is caused by biallelic germline mutations in the Mut Y human homologue (*MUTYH*) gene, encoding A/G specific adenine DNA glycosylase excision repair protein [143]. Another inherited syndrome, which follows autosomal dominant inheritance, is familial adenomatous polyposis (FAP) and is characterized by germline mutations in *APC* gene [144, 145]. With regard to risk stratification, the most robust identification strategy to date is detecting germline mutations in genes that cause these and other hereditary colon cancer syndromes (e.g., *APC* mutations in FAP and MSI and mutations in MMR genes in Lynch syndrome, *MUTYH* biallelic mutations in MAP, and *BMPR1A* in juvenile polyposis, etc.) [146]. Clinical diagnosis, which confirms the start of the disease in an individual with known hereditary mutation, is based on different surveillance schemes, which most commonly involve endoscopic imaging, such as colonoscopy or flexible sigmoidoscopy. The start and intervals of these diagnostic procedures should be based on the family history and type of mutation, as well as individual preferences (NCCN Guidelines, Colorectal Cancer Screening). In some cases, there is no known family history of the inherited syndrome; diagnosis is then based on certain histopathological characteristics of polyps and/or tumours, followed by confirmation of inherited genetic factors using genetic testing for mutations in MMR genes, *MUTYH*, and *APC*. However, hereditary syndromes account for a small percentage of colorectal cancers. About 75% of patients with colorectal cancer have sporadic disease with no apparent evidence of having inherited the disorder (NCI, Genetics of Colorectal Cancer (PDQ), 2013). The remaining 25% of cases have a family history of the disease; however, it has been estimated that well characterized genetic mutations in highly penetrant genes account for only 5% to 6% of colorectal cancer cases overall. It is likely that less penetrant inherited susceptibility genes contribute to the development of the remaining cases of familial colorectal cancer in conjunction with environmental risk factors [147]. It is well established that early screening for polyps in family members of patients with characterized mutations in MMR (Lynch syndrome), *APC* (FAP), or *MUTYH* (MAP) genes is beneficial in improving detection of malignant changes and reduces mortality. However, as sporadic colorectal cancer occurs much more frequently, it would be beneficial to establish reliable screening methods for early detection. Several developed countries successfully employ national programme screening for early detection of precancerous lesions and cancer of the colon and rectum in men and women between 50 and 69 years with the aim to reduce morbidity and mortality due to these cancers [148–150]. The most common strategy is based on using a faecal-based self-sampling kit for faecal occult blood test (FOBT), followed by colonoscopy or sigmoidoscopy, if the FOBT was positive [150, 151]. Two FOBT test are available, guaiac-based, which detects haemoglobin enzymatically, and immunochemical-based faecal immunochemical test (FIT), which detects human globin within haemoglobin. However, main limitations of these noninvasive tests are low specificity and sensitivity [152, 153]. Furthermore, enzymatic FOBT is susceptible to nonhuman heme from dietary sources and blood from upper gastrointestinal tract [153]. Although the examination by colonoscopy is expensive and inconvenient, it is still the most used type of colorectal cancer detection [153, 154].

Discovery of more suitable and noninvasive molecular biomarkers and development of reliable biomarker assays are long and complex processes [154]. Ideally, the biomarkers for early detection of colon malignancies should be shed by tumour cells and released into either bloodstream or intestinal lumen, so they could be detected in blood or faeces [155]. Several studies and clinical trials addressed identification and validation of molecular biomarkers in tumour tissues; however, tissue samples are not always available, and the quality of tissue storage varies between laboratories, therefore making validation of these markers difficult [156].

CEA, carbohydrate antigen CA 19-9, and tissue inhibitor metalloproteinase type I are the best characterized serum prognostic biomarkers to date; however, none of them is specific for colorectal cancer [153, 154, 157–159]. Five-serum-marker panel, including spondin-2, DcR3, Trail-R2, Reg IV, and MIC1, six SELDI peaks, corresponding to ApoC1, C3a-desArg, *α*_1_-antitrypsin, and transferrin, PSME3, NNMT, CRMP-2, defensins (HNP 1–3), MIF, macrophage colony-stimulating factor (M-CSF), tumour pyruvate kinase isoenzyme type M2 (M2-PK), prolactin, CCSA-2, -3 -4, metalloproteinases MMP-9 and MMP-7, and laminin have been included in preclinical validation and assay development [153, 160–162]. Unfortunately, none of the markers showed adequate specificity and sensitivity, although, M-CSF, for example, could be used for lymph node metastasis prediction [163]. Furthermore, the biological relevance of most of these biomarkers to colorectal cancer remains unclear and raises the question whether their appearance in serum could be due to secondary effects of cancer rather than specific to tumour tissues due to secretion or leakage [155]. Several other studies have assessed the predictive and prognostic significance of serum proteome from patients with colorectal cancer using MS-based proteomic methods with varying results [152, 164–169]. Some of these studies identified only peptide/protein peaks which were differentially expressed between colorectal cancer patients and healthy controls without further identification of proteins. However, these serum proteome profiles could potentially serve as a diagnostic method for colorectal cancer screening after validation on larger populations. Klein-Scory et al. researched another approach to identify specific signature of colorectal cells [170] Using 2DE, serological screening on PDVF membranes with sera from colorectal patients and healthy controls, followed by identification of selected proteins with MALDI-MS and/or by LC-MS/MS and Western blot validation they analysed the extracellular proteome of five colorectal cancer cell lines. Aiming to discover specific patterns in the secretome (extracellular proteome), which presumably triggers immune response in cancer patients and is believed to be enriched in biomarkers due to the humoral immune response, they identified two novel biomarkers, Glod4, a glyoxalase-domain containing protein, and a C-terminal fragment of agrin, a heparansulfate proteoglycan resident in basement membranes, and several other differentially expressed proteins or protein fragments, such as PGAM1, syntenin, aldolase C, LMAN2, VIP36, and Rad23b. Another interesting approach involves detecting mutant tumour proteins in the serum or tissues of colorectal cancer patients using immunoprecipitation or gel-based methods for enrichment of tumour specific biomarkers, followed by LC-MS/MS and targeted mass spectrometry approach, such as selected reaction monitoring (SRM) MS or multiple reaction monitoring (MRM) MS [171–174]. Ruppen-Canas et al. and Wang et al. used immuno-LC-MS-SRM approach to detect wild-type and mutant RAS proteins in colorectal and pancreatic cancer tissues, benign skin tumours, and pancreatic cyst fluid [173, 174]. One of the advantages of this approach is that numerous independent proteins could be assessed simultaneously in a relatively small amount of clinical samples. Identification of mutant proteins in clinical samples could potentially serve as drug-related biomarkers and aid in selection of appropriate chemotherapy strategies. Lumachi et al. used simple multianalyte immunoassay for measurement of five markers, CEA, CA 19-9 and 72-4, CYFRA 21-1, and osteopontin, comparing their expressions in patients with colorectal cancer and age- and sex-matched patients suffering from confirmed benign colorectal diseases (controls) [175]. Single marker measurements showed, as expected, low specificity and sensitivity; however, simultaneous measurements of all five markers achieved 74.1% sensitivity and 94.3% specificity in patients with colorectal cancer. The authors speculated that this panel could have enough diagnostic accuracy to be evaluated as a tool for screening for colorectal cancer.

Recent methodological advances have allowed the proteome identification of faecal-borne biomarkers, thus providing an opportunity to establish other noninvasive diagnostic methods for detecting colorectal cancer [155]. Karl and colleagues evaluated expression of S100A12 and TIMP-1 using ELISA, which showed comparable diagnostic performance with the established immune-FOBT [176]. The combination of S100A12, immune-FOBT, and TIMP-1 reached sensitivity greater than 80% at a high specificity (98%), giving noninvasive colorectal cancer screening in stool a new perspective. M2-PK was also extensively evaluated as potential secreted biomarker in stool samples by several groups of researchers with varying results [177–182]. The limited specificity of many ELISAs, and in many cases limited antibody availability for assay development and validation, and low specificity and sensitivity of established assays, coupled with recent advances in MS technology, are stimulating further research in order to obtain reliable protein or peptide profiles from faeces [183]. Ang et al. validated 60 potential secreted biomarkers selected from the literature using SDS-PAGE separation followed by MRM analysis on high pressure LC-MS system (HPLC-MS) [183]. Myeloperoxidase, hemoglobin, protein S100A9, filamin A, and L-plastin were confirmed to be present at significant levels only in the feces of the colorectal cancer patients. In another small-scale screening Ang and colleagues identified nine proteins, a-1-antityrpsin, a-1-acid glycoprotein, complement C3, fibrinogen, haptoglobin, hemoglobin *α*, hemoglobin *β*, myeloblastin, and transferrin, which were detected only in the samples from patients with colorectal cancer [184]. Initially, they used discovery approach to select peptides for analytical MRM assay. They obtained a peptide library containing 108 proteins present in faeces of both cancer patients and nondiseased patients using three alternative prefractionation strategies (SDS PAGE, reverse phase high pressure LC (RP-HPLC), and size exclusion chromatography) followed by RP-HPLC MS/MS identification. 40 proteins were selected for multiplex screening by MRM-based assay on a set of colorectal patients and healthy volunteers. Although the method is complex, the protein panel, if evaluated on a larger cohort of samples, could be reengineered to antibody-based assay, compatible with current clinical analysers [184]. Bosch et al. [185] performed a pilot study by gel electrophoresis separation and LC-MS/MS in stool samples from colorectal cancer patients and patients with negative colonoscopy. They identified 134 significantly overexpressed proteins in samples of colorectal patients.

### 2.5. Gastric Cancer

Gastric cancer is the fourth most common malignancy and the second leading cause of cancer death worldwide [58]. It is more common in developing countries. Environmental and behavioural risk factors are very important: salt intake, tobacco use, and alcohol consumption intake have a negative impact, while fruit and vegetable intake and increased use of refrigeration of food storage (rather than salting, pickling, and smoking) act protective [186]. From infectious causes, *Helicobacter pylori* is recognized as WHO class I carcinogen, and Epstein-Barr virus has also been associated with gastric cancer. There are also some known hereditary components [186]. Hereditary diffuse gastric cancer stands for early onset of diffuse gastric adenocarcinoma, autosomal dominant disease penetration, and an increased risk of lobular breast cancer and signet ring cell colon cancer. It is caused by a germline mutation in *CDH1*, a gene coding for E-cadherin, a calcium dependent cell dependent cell adhesion protein responsible for cell-cell interaction and cell polarity. It is possible that the lack of immunohistochemically detectable E-cadherin expression may be a useful diagnostic adjunct [187]. Prophylactic gastrectomy in such individuals can be performed. There are also other hereditary conditions associated to gastric cancer, such as Lynch syndrome, FAP, and Li Fraumeni syndrome [186]. The most common form of gastric cancer is gastric adenocarcinoma, which has historically been divided into diffuse and intestinal type, but there are also other classifications, that is, according to the location [186, 188].

Radical surgery is currently the only possible cure for gastric cancer, invading the muscular layer; however, the majority of patients are diagnosed at an advanced stage, where a systemic spread of the tumour cells has to be anticipated [189]. Finding early diagnosis biomarkers is therefore of utmost importance.

Unfortunately, early gastric cancer is asymptomatic. Current clinical diagnostic biomarkers, such as CEA, CA 19-9, and CA 72-4, are not specific and sensitive enough [190]. Proteomic approaches facilitated the search of new potential biomarkers and gave quite some candidates up to now. Lin and colleagues summarized some of these studies performed on different clinical samples, such as blood, gastric fluid and tissues, as well as cell lines and animal models [190]. Complement factor I precursor (CFI), C9, IPO-38, inter-*α*-trypsin inhibitor H3 (ITIH3), SLe, ApoC1, ApoC3, thrombin light chain A, and MIF were detected and validated as differentially expressed in blood specimens. Pepsinogen C (PGC), pepsin A, *α*-defensin, *α*_1_-antitrypsin precursor and *α*_1_-antitryspin, gastrokine-1 (GKN1), trefoil factor 1 (TFF1), and pepsinogen II were identified and confirmed as differentially expressed in gastric fluid. The majority of potential biomarkers were determined in paired tissue samples. Serum-binding protein 1, ENO1, glucose regulated proteins GRP78 and GRP94, cyclosporine A-binding-protein (PPIA), peroxiredoxin-1 (PRDX1), phosphatase and tensin homolog (PTEN), MAWD-binding protein; mitotic arrest-deficient 1-like 1 (MAD1L1), HSP27, protein CYR61, chloride intracellular channel 1 (CLIC1), cathepsin B, GKN1, ATP-dependent RNA helicase DDX39, lactate dehydrogenase A (LDHA), pyruvate dehydrogenase B (PDHB), hypoxia-inducible factor (HIF), cysteine-rich intestinal protein 1 (CRIP1), *α*-defensin-1, *α*-defensin-2, proteins S100A8, S100A6 and S100A9, PGC, human neutrophil peptides 1–3 (HNPs 1–3) and macrophage migration inhibitory factor, galectin-2, ApoA1, S100P, laminin gamma 2 chain monomer, human epidermal growth factor receptor 2 (HER2), glycolipid and GM2 were detected and validated as differentially expressed in tissue samples.

Our laboratory is also trying to find potential biomarkers for gastric cancer: using 2DE and MS, 39S ribosomal protein L12 (mitochondrial precursor; MRPL12), among others, was determined as under-expressed and the finding was validated with immunoblotting [191]. Analyzing paraffin-embedded samples, Sousa and colleagues found a variety of proteins overexpressed in metaplasia and intestinal type cancer: lactotransferrin (LTF) and deleted in malignant brain tumor 1 (DMBT1) were validated as metaplasia biomarkers as well as potential prognostic gastric cancer biomarkers [192]. Leucine-rich repeat-containing G protein-coupled receptor 5 (LGR5) was also determined as overexpressed in gastric cancer by immune-staining and proposed as a possible prognostic biomarker [193], and similar findings were confirmed for kallikrein 12 (KLK12) [194], and plasma membrane protein solute carrier family 3 member 2 isoform b (SLC3A2) [195]. Using plasma samples, signal peptide-CUB-EGF domain-containing protein 1 (SCUBE 1) was found elevated in patients with gastric cancer [196]. Another approach used a panel of potential serum biomarkers; epidermal growth factor receptor (EGFR), pro-ApoA1, ApoA1, transthyretin (TTR), regulated upon activation, normally T-expressed and presumably secreted (RANTES), D-dimer, vitronectin (VN), interleukin-6 (IL-6), *α*-2 macroglobulin, C-reactive protein (CRP), and plasminogen activator inhibitor-1 (PAI-I) were selected as classifiers in the two algorithms that accurately differentiated between the majority of gastric adenocarcinoma and control serum samples [197]. Taking the advantages of glycoproteomics into account, Uen and colleagues found several Con A bound glycoproteins in plasma of gastric cancer patients [198]. After validation on tumour and nontumour tissue samples, leucine-rich alpha-2-glycoprotein (LRG1) and inter-alpha-trypsin inhibitor heavy chain H13 (ITIH3) were determined as being overexpressed in tumour tissues; however, their practical use in the clinical environment is questioned. Urine is another easily accessible fluid and a possible biomarker source; endothelial lipase (EL) was found significantly decreased in urine samples of gastric cancer patients [199]. Interestingly, it was not as distinctive in tissue and serum samples.

### 2.6. Protein Biomarkers in Detection of Other Cancers

Primary brain tumours are rare in adults, but not in children [200]. Neuroepithelial tumours are the most common, and the WHO subdivides them into a variety of histologic tumour types, such as glial (astrocytomas, oligodendrogliomas, and ependymomas), neuronal, mixed glial-neuronal, embryonal, pineal, and choroid plexus derived. Astrocytomas are a morphologically heterogeneous group of neoplasms and defined as tumours with predominantly astrocytic differentiation. Diffusely infiltrating astrocytic tumours are the most common primary neoplasms in adults and constitute more than 60% of all brain tumours. Glioblastomas (WHO grade IV) are the most malignant tumours within this spectrum and account for approximately 12% to 15% of all intracranial neoplasms and up to 60% of all astrocytic tumours. Despite distinct progress in surgical resection, radiation, and chemotherapy, the prognosis of patients with glioblastoma multiforme is still very poor: 5-year survival rate is approximately 5% [201, 202]. Up to now, no established clinical cerebrospinal fluid or serum markers exist and histopathological analysis of the tumour tissue is mandatory for a definite diagnosis [202]. Proteomics enabled the identification of several promising candidates from tissue, blood, cerebrospinal fluid, cell lines or even animal models using 2DE and MS, SELDI-TOF, protein microarrays, LC-MS/MS, ELISA, and so forth [203–205]. In our laboratory we took an interesting approach of finding new potential glioblastoma biomarkers using llama heavy-chain antibodies (project GLIOMA). Camelids have two types of antibodies: regular and the so-called heavy-chain antibodies, which lack a light chain and as a consequence their antigen-binding site is reduced to one single domain (VHH or nanobody) [206]. Their sequence and structure adaptation enables them to have additional diversity in their antigen-binding repertoire, which could enable us to find either novel or altered proteins as biomarker candidates.

Oral cancer accounts for 2-3% of all malignancies and occurs most commonly in the form of squamous cell carcinoma [207, 208]. The five-year survival rate of patients is about 40% and has not improved significantly in recent decades, despite advances in surgery, radiotherapy, and chemotherapy [207, 209]. There are currently no effective methods to screen for oral cancer [207]. Saliva represents an easily obtainable clinical sample for biomarker detection; however, its protein complexity makes identification of potential biomarkers challenging [208]. One of the advantages of saliva is also the assumption that proteins and/or cells are almost certainly shed or secreted into it from oral lesions [208, 210]. Proteomic approaches based on ELISA identified potential saliva biomarkers in patients with oral cancer, such as underrepresentation of secretory immunoglobulin A, 8-oxoguanine DNA glycosylase, phosphorylated-Src, and mammary serine protease inhibitor (Maspin) and overrepresentation of insulin growth factor I, metalloproteinases MMP-9, MMP-2, CD44, cytokeratin 19 fragment, tissue polypeptide antigen, CA125, and Cyclin D1 [211–215]. Mass spectrometry-based studies indicated differential expression of several proteins [209, 216]. The most promising for early identification of oral malignancy could be a panel of five candidate biomarkers (M2BP, MRP14, CD59, profilin, and catalase) or a panel of six proteins (keratin 6B (ck6) and 13 (ck13), *β* globin, *α*-2-actin, HSP70, and HSP90) [209, 216]. Kooren and colleagues showed increased relative abundance of six proteins (hnRNPM, IL1F6, LCN2, S100A8, NQO1, and XRCC5/6) in brush biopsies from the patients with dysplastic oral premalignant lesions compared to saliva collected from the same patients [208].

## 3. Conclusions and Future Perspectives

It is now commonly acknowledged for several cancers that early detection benefits saving lives. Novel, nanotechnology-based, ultrasensitive immune-sensors hold promise to revolutionize cancer detection, monitoring, and therapy in the future with the ability to measure panels of specific, selective cancer biomarker proteins on-the-spot in physicians' surgeries and clinics, together with the development of more accurate proteomic methodologies for clinical laboratory setting [5]. Protein biomarkers, especially when detected in easily accessible clinical samples, such as blood, faces, urine, oral swabs, or saliva, could be of great use in this field. However, the sensitivities and specificities of currently used protein biomarkers are usually too low to be used in clinical setting. It is now well accepted that a single biomarker will not suffice; rather a panel of proteins is necessary to aid in diagnostics. Proteomic studies gave several candidates up to now and with a large-scale validation an appropriate combination could be determined.

As can be evident from numerous studies on biomarker discovery for different cancers, several obstacles still hold back translation of research into routine clinical practice. Despite advances in MS-based methods and extensive research, there is still no sensitive and specific serum biomarker panel, which could be used for diagnostic or screening purposes. Discovery and validation of suitable protein biomarkers for noninvasive screening and diagnostics are further impeded by the dynamic complexity of blood, serum, or faeces proteome. Another problem is validation of novel biomarkers and obtaining large cohorts of patients; however, even accumulation of data performed in different research laboratories could eventually contribute to the validation process through meta-analyses. Heterogeneity of sporadic cancers implies another obstacle to overcome. The biomarker studies mostly search for novel protein signatures in highly defined patient populations and still different protein changes in individuals across different patient populations are found, which further limit the translation of discovered biomarkers into clinical setting. For general screening purposes, multicentred large studies and meta-analyses should be performed in order to improve the accuracy and sensitivity of the test.

## Figures and Tables

**Table 1:** Selection of commonly used blood molecular and protein biomarkers for the management (diagnosis, monitoring recurrence/detecting metastatic spread, and selection of suitable treatment/monitoring treatment response) of cancers in clinical practice.

Type of cancer	Tumour biomarkers	Type of test	Additional biomarkers	Type of test
Lung cancer	NSE, TPA, and CEA (large-cell carcinoma, small-cell carcinoma) CEA, TPA (adenocarcinoma) SCC, TPA (squamous cell carcinoma)	Immunoassay, ELISA, RIA, and magnetic-bead based chemiluminescence enzyme immunoassay	Ferritin	Immunoassay, ELISA, and RIA
			CEA	

Breast cancer, sporadic	CA 15-3, CA 27-29, MCA, and CEA	Immunoassay, ELISA	TPA, B2M, Ki-67 (MIB-1), CA 19-9, ER, PR, and cytochrome P450 *2D6* genotype	Immunoassay, PCR-based assay for cytochrome P450
	HER2*	Immunoassay, fluorescence in situ hybridization (FISH)		

Breast cancer, hereditary	BRCA1, BRCA2	DNA sequence analysis, RT-PCR, and post-PCR curve melting analysis	The same as for sporadic breast cancer for monitoring	Immunoassay

Prostate cancer	PSA, PAP	Immunoassay, ELISA	CEA, TPA	Immunoassay

Gastric cancer, sporadic	CA 19-9, CA 125, and CEA HER2*	Immunoassay, ELISA	TPA, ferritin, and gastrin	Immunoassay
		Immunoassay, fluorescence in situ hybridization (FISH)		

Colorectal cancer, hereditary	MSI testing (BAT25, BAT26, NR-21, NR-24, and MONO-27), APC, AXIN2, BMPR1A, CDH1, CHEK2, MLH1, MLH3, MSH2, MSH6, MYH/MutYH), TP53, PTEN, PMS2, SCG5/GREM1, SMAD4, and STK11	PCR-based test, IHC, DNA sequence analysis, long-range PCR, RT-PCR and post-PCR curve melting analysis, multiplex ligation probe amplification (MLPA), and array comparative genomic hybridization (aCGH)	The same as for sporadic colon cancer for monitoring	Immunoassay
	BRAF, MLH1 promoter methylation	PCR-based test		

Gastric cancer, sporadic	CA 19–9, CA 125, and CEA HER2*	Immunoassay, ELISA	TPA, ferritin, and gastrin	Immunoassay
		Immunoassay, fluorescence in situ hybridization (FISH)		

Gastric cancer, hereditary	CHD1	DNA sequence analysis, RT-PCR, and post-PCR curve melting analysis	The same as for sporadic gastric cancer for monitoring	

Oral cancer	SCC, CEA	Immunoassay	CA 19-9	Immunoassay

Glioma	MGMT promoter methylation	Methylation specific PCR	—	—
	IDH1, IDH2	DNA sequence analysis, RT-PCR, and post-PCR curve melting analysis	—	—

*FISH using labeled DNA probes to the pericentromeric region of chromosome 17 and to the HER2 locus is superior to Southern, Northern, and Western blots and immunohistochemical analyses. Usually performed on tissue, however, blood tests can be ordered. (Note: individual laboratories may use different or other biomarkers and tests.)

## Abbreviations

2DE: Two-dimensional gel electrophoresis
2D-DIGE: Difference gel electrophoresis
CBB: Coomassie Brilliant Blue
CIMP: CpG island methylator phenotype
ESI: Electrospray ionisation
FAP: Familial adenomatous polyposis
ICAT: Isotope-coded affinity tag
IEF: Isoelectric focusing
iTRAQ: Isobaric tags for relative and absolute quantitation
LC: Liquid chromatography
MALDI-TOF: Matrix-assisted laser desorption/ionization time-of-flight
MAP: MUTYH-associated polyposis
MMR: Mismatch repair
MS: Mass spectrometry
MS/MS: Tandem mass spectrometry
MSI: Microsatellite instability
pI: Isoelectric point
PTM: Posttranslational modification
SDS-PAGE: Polyacrylamide gel electrophoresis
SILAC: Stable isotope labelling by amino acids in cell culture.
